# Effect of the biomass production conditions on survival of the probiotic yeast *Saccharomyces cerevisiae* var. *boulardii* in a laboratory model of the gastrointestinal tract

**DOI:** 10.1186/s12934-026-02999-8

**Published:** 2026-04-12

**Authors:** Dorota A. Rzechonek, Lisbeth Olsson

**Affiliations:** Department of Life Sciences, Division of Industrial Biotechnology, Chalmers University of Technology, Chalmersplatsen 4, 412 96 Gothenburg, Sweden

**Keywords:** Probiotics, *Saccharomyces boulardii*, Biomass production, Acetic acid

## Abstract

**Background:**

Saccharomyces *cerevisiae* var. *boulardii* is a commercialized probiotic yeast used for the treatment of diarrhea and other disorders of the gastrointestinal tract. While the impact of this yeast on the body and its interaction with other microorganisms is well studied, little information about the production of probiotic yeasts is publicly available. This study aimed to improve the performance of *S. boulardii* by optimizing yeast biomass production.

**Results:**

We developed a simple in vitro model that mimicked the conditions of the gastrointestinal tract. The model allowed us to detect differences in performance between yeast produced under different conditions. The three-step set-up consisted of incubations in stomach-like and small intestine-like juice, followed by cultivation under colon-simulating conditions with intestine-like media (ILM) at varying pH. *S. boulardii* strains were initially unable to growth in ILM at pH 7.0. However, by optimizing production conditions, we obtained yeast capable of surviving the whole pH range in ILM. Yeast biomass displaying the best performance was harvested in early stationary phase from cultures grown at 37 °C in YPD medium. Moreover, during cultivation under colon-like conditions, we detected acetic acid, which is a desirable feature of probiotic microorganisms.

**Conclusion:**

The choice of production conditions is crucial for the quality and functionality of probiotic yeast biomass. Differences in biomass viability can be observed by applying a model of the gastrointestinal system, which can be a useful tool in further studies on probiotic yeasts.

**Supplementary Information:**

The online version contains supplementary material available at 10.1186/s12934-026-02999-8.

## Background

*Saccharomyces cerevisiae* var. *boulardii* is a subspecies of the yeast *S. cerevisiae*. Strains of *S. boulardii* have been isolated in Southeast Asia from mangosteen, lychee, and pineapple fruits, which were traditionally used as a remedy for diarrhea [1]. Some strains, such as CNCM I-745, have already been commercialized. To achieve the expected health benefits of yeast-based probiotics, these preparations must be of the highest quality. Choice of production methods is an important factor in the final product quality, however information on this process is not publicly known.

*S. boulardii* is applied in the treatment and prevention of diarrhea, relief from symptoms of chronic gastrointestinal diseases, and support in antibiotic therapy [1]. It is particularly recommended against diarrhea caused by the bacterium *Clostridium difficile.* Yeast is thought to facilitate pathogen inactivation by binding to their cell wall and releasing proteases that can degrade toxins [2]. *S. boulardii* can also produce acetic acid [3]. This and other short-chain fatty acids exert beneficial health effects by strengthening gut barrier function, modulating immune responses, and affecting the brain-gut axis [4].

As probiotics, yeast cells are subjected to the diverse and challenging conditions encountered in the human body. The first challenge is a temperature of 36.6 °C, which is higher than the 28–30 °C optimal for most yeast species. After ingestion, the cells must survive the very low pH of the stomach (pH 1–2 when it is empty [5]), while the pH optimum for *S. cerevisiae* is 4 to 6 [6].

Next, cells are moved to the duodenum, the first section of the small intestine, where the pH rapidly augments following the inflow of pancreatic juice from pancreatic and bile from the gall bladder. The high concentration of bile salts poses a challenge for yeast, however 95% of bile salts are reabsorbed before the digestive contents continue to the large intestine [7].

The large intestine is populated by billions of microorganisms, the presence and diversity of which is crucial for human health. While it is the expected site of action for most probiotics, it is still not the optimal environment for yeast. For one, access to simple carbohydrates is limited. The available carbon sources are complex polysaccharides and proteins, whose breakdown involves the formation of by-products [5]. In addition, the pH is closer to neutral, while yeast prefer a slightly acidic milieu [8].

*S. boulardii* is thought to tolerate very low pH and elevated temperatures better than other *S. cerevisiae* strains, making it suitable as a probiotic. In addition, it switches to pseudohyphal growth easier [1], which can provide better adherence to intestinal epithelial cells. Despite this advantage, *S. boulardii* does not permanently colonize the gastrointestinal tract of mammals. Its cells are no longer detected in human feces 3–5 days after termination of probiotic treatment [9], echoing similar findings in mouse models [10]. However, fungemia cases associated with probiotic therapy have been described in severely immunocompromised patients, including fatal outcomes [11, 12]. To reduce the risk of fungal infection associated with probiotic treatment, heat-killed *S. boulardii* can be used, as it still alleviates symptoms of some diseases such as ulcerative colitis [13, 14].

Studies on *S. boulardii* often consider the stress triggered by low pH in the stomach or the presence of bile salts in the small intestine; however, they rarely address the effects of varying pH in the large intestine. The pH rises along the length of the large intestine, starting at around 6.0 in the caecum and finishing at 7.2 in the descending colon [15]. However, pH values are affected by several factors, including gender, diet, age or health of the individual and can range from 5.2 to as high as 8 [5].

Anomalies in colonic pH have been documented across various pathological states. An alkaline environment indicates an improper balance of microorganisms in the microbiome and encourages infections such as those caused by *C. difficile* [16]. Furthermore, an elevated fecal pH exceeding 6.6 has been identified as a significant prognostic indicator associated with increased mortality in critically ill patients [17]. On the other hand, pH lower than 5.5 may be associated with malabsorption and the subsequent overproduction of organic acids. It has also been observed in some patients suffering from Inflammatory Bowel Disease (IBD) or Irritable Bowel Syndrome (IBS) [16].

The production of yeast probiotics is another topic that has been rarely addressed in the scientific literature. Some information can be found in patents, but usually production technologies are not disclosed by companies. Meanwhile, the specific method can be crucial to the quality and functionality of probiotics, as observed for bacterial species *Lactococcus lactis* [18] or bifidobacteria [19].

In *S. cerevisiae*, efforts to optimize cell production have focused chiefly on subsequent ethanol fermentation [20]. Stepwise introduction of short-term adaptations significantly improved production parameters even on substrates as demanding as lignocellulosic hydrolysates [21]. These results raise hopes that a similar approach could unlock the full potential of probiotic properties in *S. boulardii*.

The aim of the present study was to investigate how production of yeast biomass affected the performance and robustness of probiotic yeast cells. Probiotic products should be able to survive in the gastrointestinal tract, affect microbiome, and have an overall positive impact on human health. In the present work, we focused on the ability of yeast produced under different conditions to survive and proliferate in an in vitro model of the gastrointestinal tract that included the varying environment of the colon. We identified a significant knowledge gap regarding the effects of near-neutral pH on *S. boulardii*—a factor that may be decisive for the probiotic’s survival and its overall interaction with the host.

## Methods

### Strains

We used four strains of *S. cerevisiae* var. *boulardii*: MYA-796, MYA797, CNCM I-745, and NCYC3264. Strains MYA-796 and MYA-797 were purchased from the ATCC strain collection (Manassas, VA, USA), while NCYC3264 was purchased from the National Collection of Yeast Cultures (Norwich, UK). CNCM I-745 was isolated from ENTEROL, a commercial probiotic preparation (Biocodex, Gentilly, France) and will be referred to as Biocodex strain. Two commonly used laboratory strains of *S. cerevisiae*, CEN.PK113-7D (later described as CEN.PK) from Scientific Research and Development GmbH (Oberursel, Germany) and S288c obtained from University of Milano-Bicocca, were also included in the study.

### Media

YPD liquid medium, consisting of 10 g/L yeast extract (VWR, Radnor, PA, USA), 20 g/L glucose (Thermo Fisher Scientific, Waltham, MA, USA), and 20 g/L peptone (GIBCO, Grand Island, NY, USA), was used for inoculum preparation, biomass production, and storage. YPD agar plates (20 g/L agar) were used for short-time storage.

Delft medium, consisting of 20 g/L glucose (Thermo Fisher Scientific), 5 g/L (NH_4_)_2_SO_4_, 3 g/L KH_2_PO_4_, 1 g/L MgSO_4_·7H_2_O, trace element solution (1 mL for 1 L medium), and vitamin solution (1 mL for 1 L medium) [22], was adjusted to pH 5 with KOH and potassium hydrogen phthalate (final concentration of 20 mM) and used for biomass production.

Several variations of intestine-like medium (ILM) were used in the study. All of them contained 5.7 g/L Bacto™ tryptone (GIBCO), 2.4 g/L glucose (Thermo Fisher Scientific), 6.4 g/L NaCl, 0.68 g/L KH_2_PO_4_, 0.3 g/L NaH_2_PO_4_, and 1 g/L NaHCO_3_. Full ILM [23] included also 5.6 g/L bile salts containing a 1:1 mixture of sodium cholate and sodium deoxycholate (Sigma-Aldrich, St. Louis, MO, USA), 0.2 g/L lysozyme (Roche, Mannheim, Germany), 1000 U/L α-amylase, 110 U/L trypsin, 380 U/L α-chymotrypsin, and 960 U/L lipase (all Sigma-Aldrich). ILM was adjusted to pH 7.5 with NaOH, mimicking the conditions in the duodenum.

For conditions resembling the colon, ILM batches with a pH range of 4.5 to 8.0 were prepared. The pH was adjusted by adding HCl or NaOH in the absence of any buffer and the pH was recorded after filter-sterilization.

### Biomass production for initial screening

Production of yeast biomass for initial screening experiments was carried out in a volume of 5 mL in 15-mL Falcon tubes, agitated at 200 rpm in a KS 4000 i control shaker (IKA, Staufen, Germany). YPD or Delft supplemented with 2% glucose were used for screening. Biomass was harvested at 6, 18, and 42 h during fermentation at either 30–37 °C. The inoculum for biomass production was taken from a YPD agar plate, except for the 6 -h sample, whereby the inoculum was taken from an overnight liquid YPD culture.

### Biomass production in flasks

Biomass production was carried out in 30 mL YPD inside 100-mL flasks agitated at 180 rpm in an Innova 44 shaker (New Brunswick, Edison, NJ, USA). The flasks were connected to a Cell Growth Quantifier (Aquila Biolabs now Scientific Bioprocessing), which allowed real-time monitoring of growth curves by measuring backscattered light. The inoculum was taken from a YPD agar plate. Samples grown at 30–37 °C were collected after 12 h (logarithmic growth phase), 18 h (early stationary phase), and 42 h (stationary phase).

### Model of the human gastrointestinal tract

**Fig. 1:**
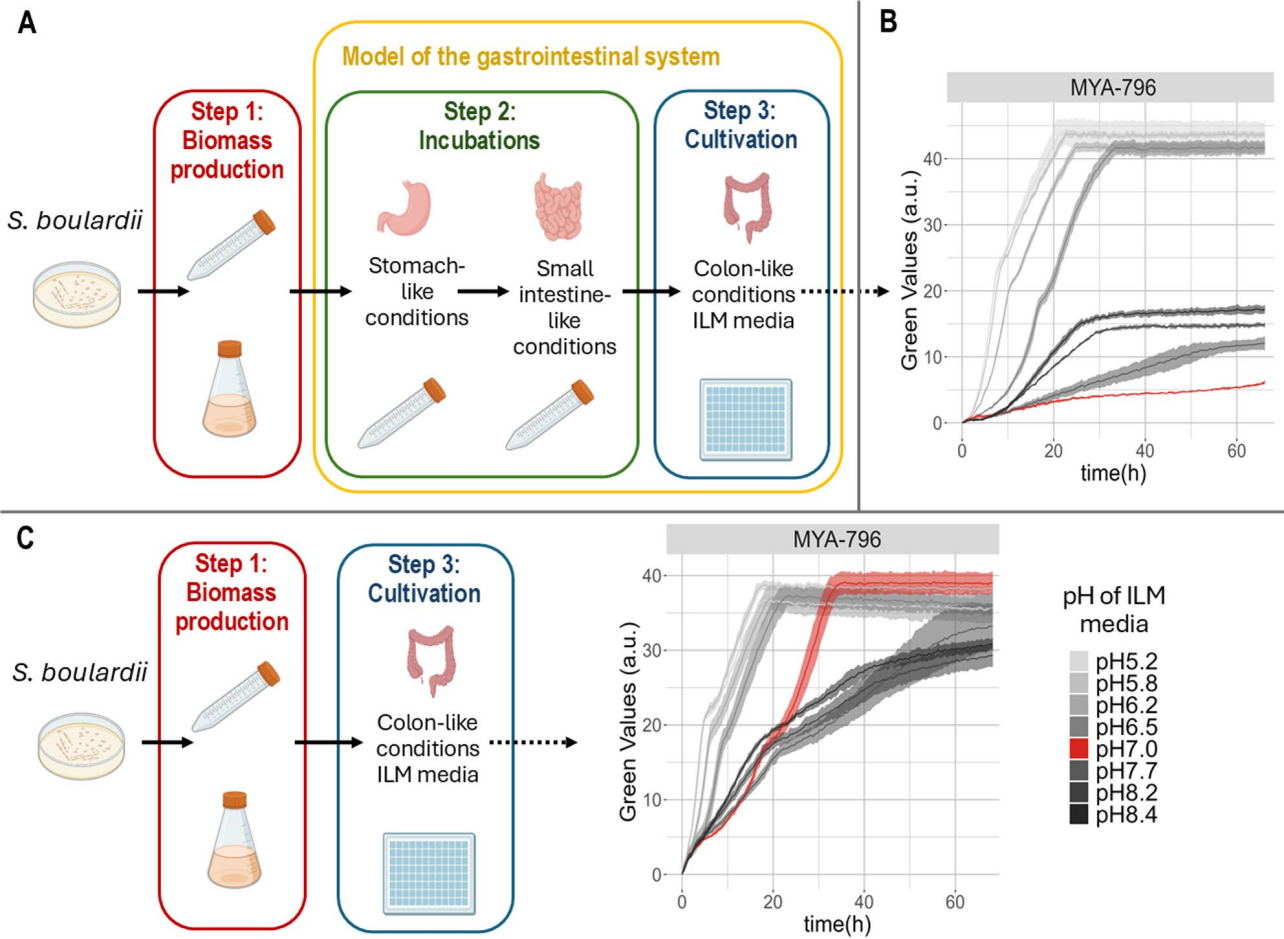
Establishing an in vitro model of the gastrointestinal tract. **A** Workflow of the experiments. **B** Growth curves of strain MYA-796 cultivated in colon-like condition (Step 3). Biomass for the experiment was produced in YPD for 18 h at 30 °C (Step 1) and then subjected to Incubations (Step2). **C** Growth curves of strain MYA-796 cultivated in colon-like condition (Step 3). Biomass source was the same as in point B, but it was not subjected to Incubations

The in vitro model of the gastrointestinal tract (Fig. 1A) consisted of three elements mimicking the conditions in the stomach, small intestine, and large intestine (colon). Cultures from the biomass production step were centrifuged at 7000 rpm for 3 min. The obtained yeast biomass was washed twice with milliQ water and then resuspended in a solution resembling stomach juice (10 mM HCl, 5 g/L NaCl, 15 mg/L pepsin, pH 2). The initial OD_600_ of samples introduced in the system was set at 2.5. Incubation in stomach-like conditions lasted 1 h at 37 °C and 200 rpm and was carried out in 2-mL Eppendorf tubes. Subsequently, 0.7 mL of the cell suspension was transferred to 3.5 mL of full ILM medium (with 5.6 g/L bile salts). After 4 h of incubation at 37 °C and 200 rpm, the samples were centrifuged at 4100 rpm for 5 min to remove the medium. The incubation periods and pH values for stomach-like and small intestinal-like conditions were selected based on physiological average transit times and pH measurements [5, 15]. Similarly, the bile salt concentration was established to reflect the mean concentrations typically found in the duodenum after a meal [7].

The yeast biomass was resuspended in ILM at pH 7.5 (without bile salts) and 50 µL of the suspension was added to wells (96-well CR1496dg; Enzyscreen, Bennebroek, The Netherlands) containing 200 µL of ILM at different pH. Each combination of strain and medium was grown in four replicates. Cultivation under colon-like conditions was carried out in a Growth Profiler 960 plate reader (Enzyscreen) at 37 °C and continuous shaking at 250 rpm. The plates were covered with lids (DC1296; Enzyscreen) minimizing access to air. Thus, after depletion of the oxygen initially present in the wells, microaerobic conditions were established. The cultivation proceeded for 70 h. For some experiments whole plates were prepared in triplicate, and the cultivation was terminated at different time points (24, 48, and 70 h) to collect samples for high-performance liquid chromatography (HPLC) and pH measurements. The parameter used to determine growth by Growth Profiler plate reader was Green Value (GV). Green Values represent the pixel intensity of light scattered by biomass, extracted from the well-bottom photos made by Growth Profiler every 30 min. The correlation between Green Values (GV) and cell dry weight (CDW) is provided in the Supplementary Data (Figure S1), along with a detailed description of how the Growth Profiler was utilized in the development of the model (Figure S2).

### Analytical methods

After the completion of Growth Profiler cultivations, the pH of the samples was measured with a FiveEasy pH meter (Mettter Toledo, Greifensee, Switzerland). Next, they were filtered and analyzed by HPLC for glucose, glycerol, ethanol, and acetate concentrations. The HPLC system was equipped with an RT-4075 RI detector, UV-4075 detector (both Jasco, Tokyo, Japan), and Rezex ROA-organic acid H^+^ column (Phenomenex, Torrance, CA, USA). Separation was performed at 80 °C with a flow rate of 0.8 mL/min and 5 mM H_2_SO_4_ as eluent.

Because of the restricted culture volumes in Growth Profiler, samples for pH and HPLC analysis were pooled from three separate wells. Consequently, the performance of independent biological replicates and subsequent statistical evaluation was constrained.

### Data processing

Data processing and analysis were performed using R (version 4.5.0) in the RStudio environment. Statistical significance was determined using Student’s T-test for pairwise comparisons. Data visualization was generated using the ggplot2 and ggpubr packages.

Screening experiment results are presented as a heatmap of normalized maximal Green Values. Normalization was performed using the following formula:

1 $$\:{x}_{\mathrm{norm}}=\frac{x-{x}_{\mathrm{m}\mathrm{i}\mathrm{n}}}{{x}_{\mathrm{m}\mathrm{a}\mathrm{x}}-{x}_{\mathrm{m}\mathrm{i}\mathrm{n}}}$$

where: $\:{x}_{norm}$ is the normalized value, $\:x$ is the original value subjected to normalization, $\:{x}_{min}$ is the minimum value within the normalized range and $\:{x}_{max}$ is the maximum value within the normalized range.

### Robustness evaluation

The maximum Green Values from all cultures in the Growth Profiler were used to estimate the robustness of *S. boulardii*. The Fano factor-based equation proposed by Trivellin et al. [22] was employed:

2 $$\:R=-\frac{{\sigma\:}^{2}}{\stackrel{-}{x}}\times\:\frac{1}{m}$$

The Fano factor is the variance (σ^2^) divided by the mean ($\:\stackrel{-}{x}$) of analyzed strains across all perturbations. It is normalized by dividing by the mean (m) of all strains. The perturbation space used for the study corresponded to the different conditions of biomass production.

## Results

### Model of the gastrointestinal tract

The first part of the study focused on the development of an in vitro laboratory model that mimicked the challenges posed by the gastrointestinal tract. The workflow consisted of three steps (Fig. 1A). Step 1 was biomass production. Yeast cells were grown under varying conditions to investigate the role of different media, temperature, growth phase or culture volume on subsequent performance in the in vitro gastrointestinal tract.

In Step 2, yeast biomass was first incubated under conditions resembling the stomach and then the small intestine. Finally, in Step 3, yeasts were cultivated under conditions resembling the colon. It was carried out in 96-well plates and monitored with a Growth Profiler plate reader. ILM media with pH ranging from 5 to 8, low in sugar but rich in amino acids, were used for cultivation. The influence of medium pH on growth in step 3 was investigated in detail, as this parameter can fluctuate in the large intestine.

Yeast biomass (Step1) was produced overnight in YPD at 30 °C. During cultivation in the in vitro model (Step 3), specific growth rates and titers varied widely depending on pH of ILM (Fig. 1B). Interestingly, *S. boulardii* strains proved to be most sensitive to neutral pH, exhibiting better growth at lower or higher pH. This might pose a problem for their use as probiotics, as neutral pH is predominant in the colon. Increased sensitivity to neutral pH was linked to incubations in stomach-like and small intestine-like environments, as growth was not impaired when Step 2 was omitted (Fig. 1C).

### Screening for the best biomass production conditions

**Fig. 2:**
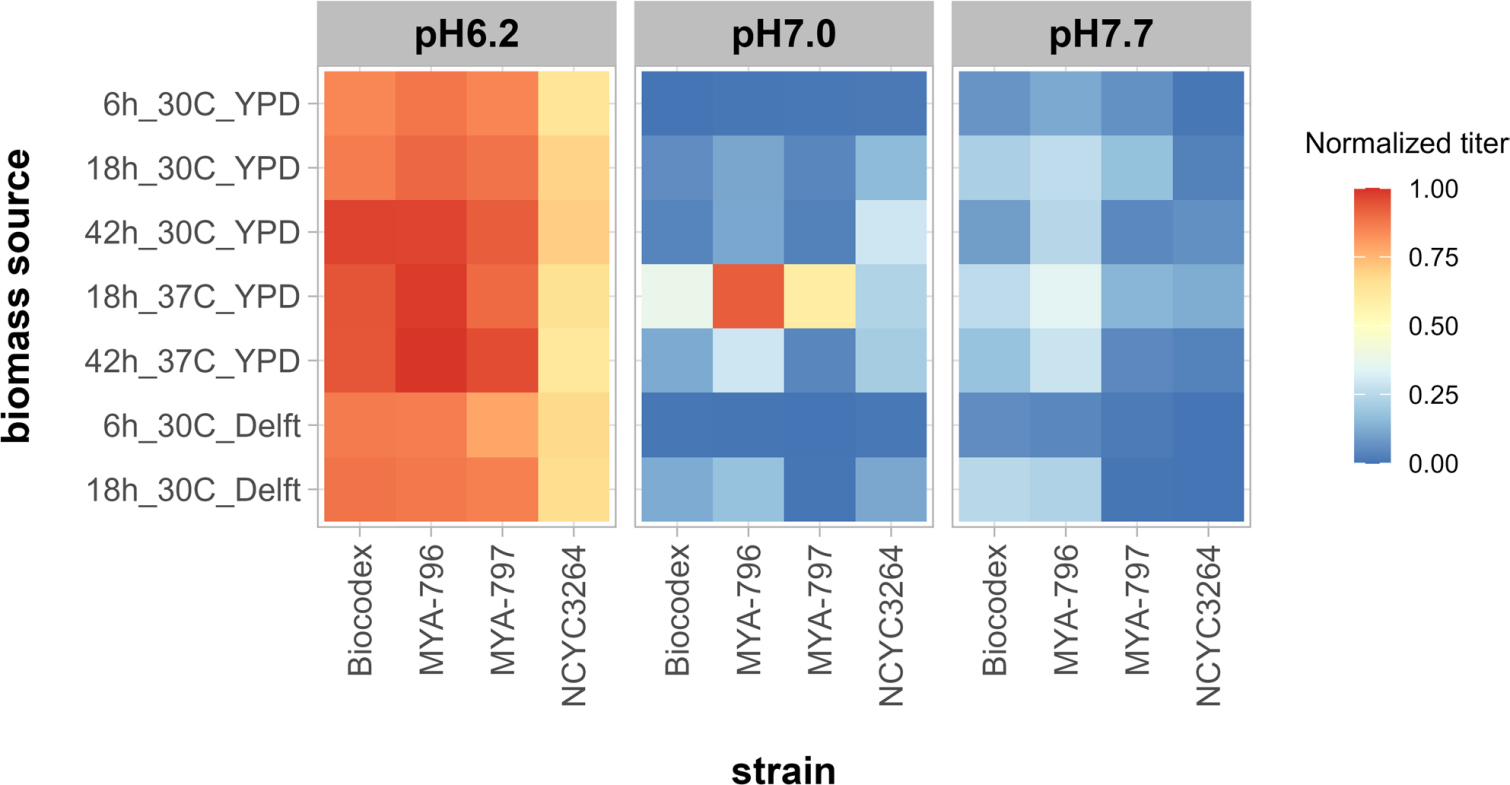
Heatmap of normalized titers (maximum Green Values) obtained during the cultivation step in ILM at pH 6.2, 7.0, and 7.7. Various strains and conditions were used to produce yeast biomass

Having set up the in vitro model, different conditions of biomass production (i.e., cultivation temperature, duration, and media) were tested. Four strains of *S. boulardii* were included: Biocodex, MYA-796, MYA-797, and NCYC3264. Figure 2 summarizes the maximum increase in biomass obtained following 66 h of cultivation in ILM at three pH values shown as normalized values (Eq. 1).

When cultured in ILM at pH 6.2, all tested strains were able to grow regardless of the biomass production method used. However, strain NCYC3264 attained lower biomass titers (maximum Green Values) than the other strains.

The influence of biomass production conditions on growth was most pronounced at pH 7. Cultures inoculated with biomass collected after 6 h (early exponential phase) were unable to grow regardless of cultivation medium. Weak growth was detected for cultures whose inoculum had been grown at 30 °C and harvested at 18–42 h. Changing the production temperature to 37 °C and harvesting biomass at 18 h led to the highest titers during the cultivation step. In particular, strain MYA-796 titer reached 44.2 ± 0.9, although extending biomass production at 37 °C to 42 h decreased the titer.

Biomass preparation conditions had no clear effect on cultures incubated in ILM at pH 7.7; although titers were significantly lower compared to those obtained at pH 6.2. Screening of production methods was performed also in Delft + 2% glucose, however, further utilization of this medium was discontinued due to generally poorer performance of obtained biomass (data not shown) and greater variability in results across different *S. boulardii* strains.

### Yeast biomass production in shake flasks

**Fig. 3:**
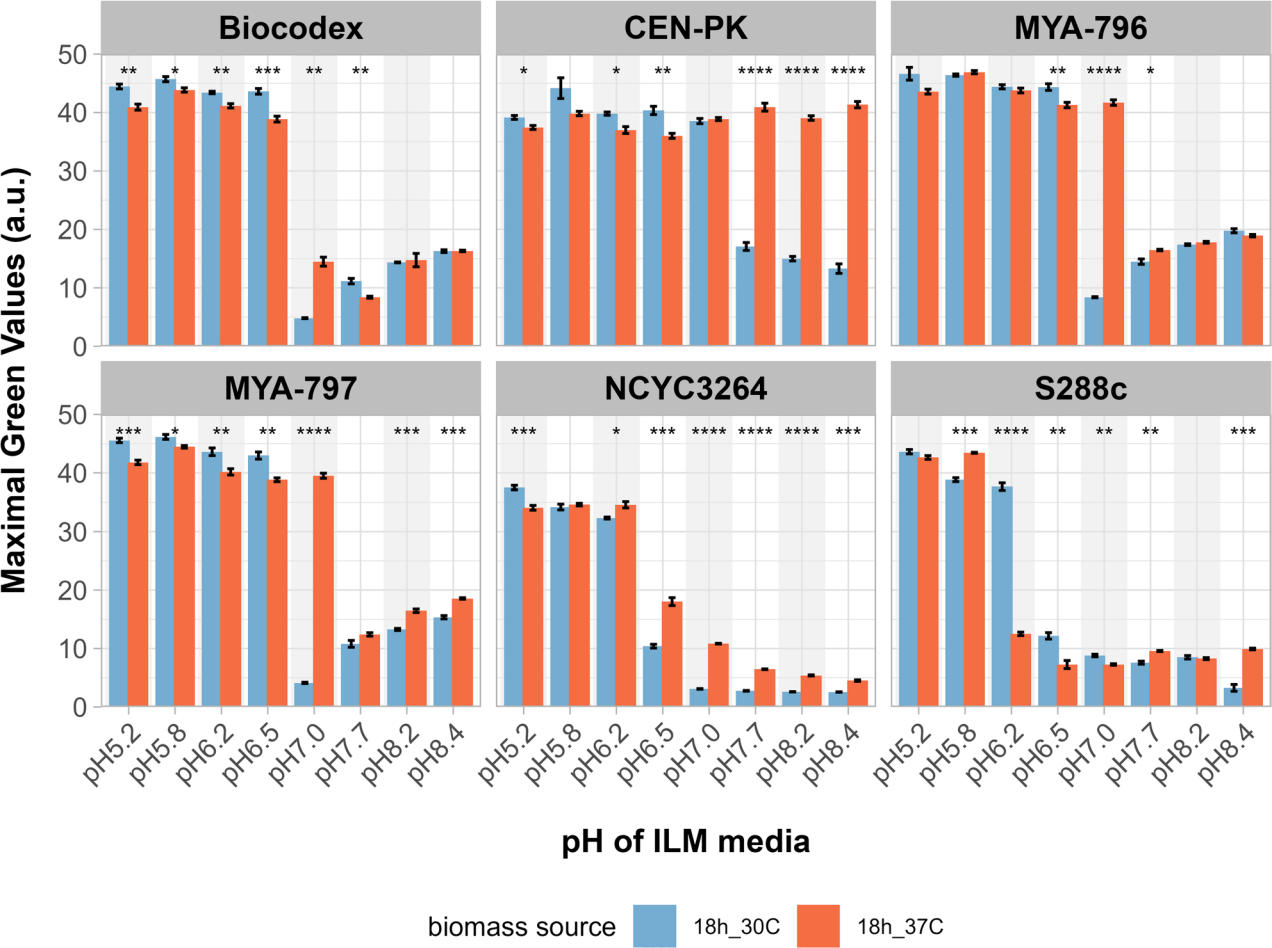
Maximum titers of tested strains achieved upon cultivation in ILM at pH 5.2–8.4 during 66 h of cultivation in Growth Profiler. Biomass for the experiment was produced at 30–37 °C and collected after 18 h. Asterisks indicate statistical significance levels between temperatures (* *p* ≤ 0.05, (* *p* ≤ 0.05, ** *p* ≤ 0.01, *** *p* ≤ 0.001, **** *p* ≤ 0.0001) based on pairwise T-tests for each pH condition

Next, we wanted a better insight into the effect of growth phase at the time of yeast biomass harvesting. Biomass production was carried out in flasks (30-mL working volume) and monitored in real time. Biomass was grown in YPD at 30 and 37 °C, and collected during logarithmic growth (12 h), at the beginning of stationary phase (18 h), and in late stationary phase (40 h). Similarly to the previous experiment, biomass grown at 37 °C and collected after 18 h out-performed biomass collected after 12 h and 40 h in the cultivation step. Biomass from the logarithmic phase (12 h) performed worst (data not shown). Here we focus on comparing biomass grown at 30 vs. 37 °C and collected at the beginning of stationary phase (18 h). Figure 3 shows the biomass titers (maximum Green Values) obtained by each of the tested strains in ILM at pH 5.2 to 8.4 during 66 h of cultivation. Additionally, we checked how the performance of *S. boulardii* compared to that of two commonly used *S. cerevisiae* strains, S288c and CEN.PK113-7D. Both strains are used as models, but they differ significantly in terms of stress-tolerance, fermentation performance, and certain physiological traits [24]. For biomass produced at 30 °C, *S. boulardii* Biocodex, MYA-796, and MYA-797 exhibited a similar pattern of pH-sensitivity during the cultivation step. At pH 5.2 to 6.5, the titers were similar and exceeded a value of 40; however, at pH 7, much lower biomass titers were observed. For strains Biocodex, MYA-796 and MYA-797, maximum Green Values were 4.8 ± 0.2, 8.4 ± 0.2 and 4.1 ± 0.2 respectively. Interestingly, at higher pH, the titers began to rise but did not exceed 20. For biomass produced at 37 °C, the pattern was similar as for 30 °C, except for a significantly higher maximal Green Values at pH 7: 14.5 ± 1.3 for Biocodex, 41.7 ± 0.9 for MYA-796 and 39.5 ± 0.9 for MYA-797.

*S. boulardii* NCYC3264 produced at 30 °C was able to grow in ILM at pH 5.2–6.5. At higher pH, the Green Values stay close to 2.4, which was the starting value at the beginning of the cultivation step. However, if the same strain was produced at 37 °C, it could grow beyond pH 7, albeit at low titers (Fig. 3B). Remarkably, the changes in growth pattern observed for NCYC3264 most closely resembled those of *S. cerevisiae* S288c.

*S. cerevisiae* CEN-PK outperformed all other strains. When biomass was prepared at 30 °C, titers exceeded 35 in ILM at pH 5.2–7.0; while at higher pH, they ranged from 17.1 ± 1.3 for pH 7.7 to 13.3 ± 1.5 for pH 8.4. Switching biomass production to 37 °C further improved titers, with maximum Green Values exceeding 35 throughout the pH range.

### Effect on the environment

Given the above results, we next investigated how yeast affected their environment during Step 3. As ILM was not buffered, it was possible to measure fluctuations in pH, which could indicate the production of potentially interesting compounds such as short-chain fatty acids. We also monitored whether any such chemicals correlated with changes in growth rate.

**Fig. 4:**
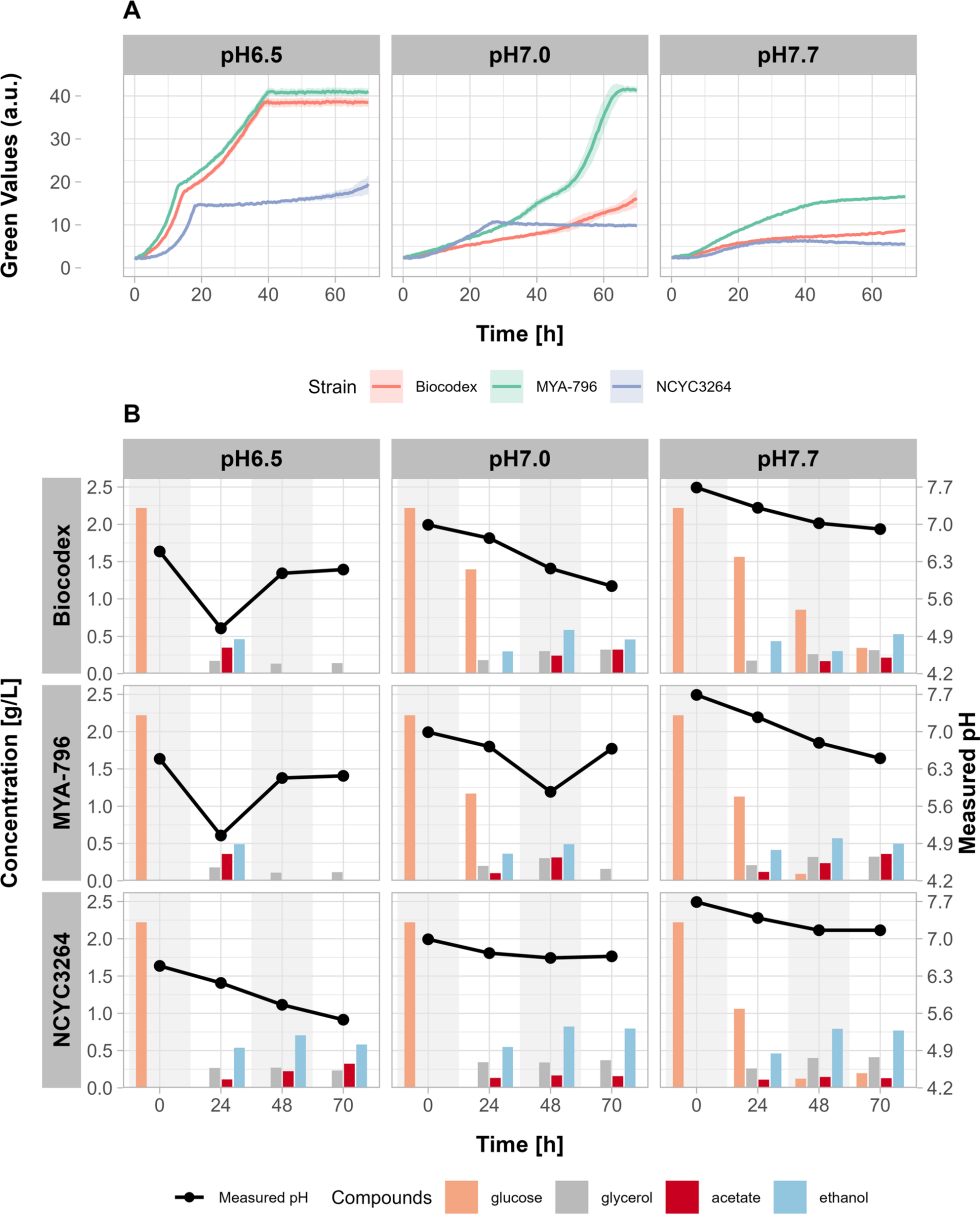
Performance of *S. boulardii* strains, during cultivation in ILM media of initial pH 6.5, 7.0, and 7.7. Biomass for the experiment was produced at 37 °C and collected after 18 h (**A**) Growth curves. **B** Changes in pH and culture broth composition of ILM media

Tests were performed with biomass produced at 37 °C for 18 h. Plates for cultivation were prepared in several replicates and run for 24–48 h. Figure 4 shows the changes in growth curves (Fig. 4A), pH, and culture broth composition (Fig. 4B) during cultivation of *S. boulardii* strains in ILM at an initial pH of 6.6, 7.0, and 7.7. Strains MYA-797 and MYA-796 behaved similarly; hence, only one is shown.

RI detector of HPLC identified three main compounds produced in ILM: glycerol, ethanol, and acetate (Fig. 4B). Measuring acetate is of particular interest as its production is considered a hallmark of probiotic potential; meanwhile, monitoring changes in ethanol and glycerol levels can provide valuable insights into the physiological state of the cells and the environmental conditions.

Glycerol was detected in cultures after 24 h in all the samples, and its concentrations did not change much thereafter. Glucose utilization was also measured, although it should be noted that peptides and amino acids from bacotrypton represent another carbon sources in the media.

The growth curves of Biocodex and MYA-796 exhibited a diauxic shift after 12 h (MYA-796) and 15 h (Biocodex) of cultivation in ILM with starting pH 6.5 reached a plateau after 40 h (Fig. 4A, left panel). Rapid growth was accompanied by a drop of pH to 5, along with the appearance of glycerol, acetate, and ethanol (Fig. 4B, left panels). Acetate and ethanol were no longer detected at 48 h in samples taken after entering stationary phase, whereas pH increased to > 6. For strain NCYC3264, the plateau was reached before 20 h (Fig. 4A, left panel), but a second increase in Green Values was noticeable after 40 h. At the same time, pH slowly decreased throughout cultivation.

The drop in pH may be explained by the accumulation of acetate and H⁺ following dissociation of acetic acid. We tested this hypothesis by adding up to 0.35 g/L acetic acid to ILM. This amount was close to the maximal concentrations detected during cultivation. In all media, the addition of acetic acid caused a significant drop in pH, albeit slightly smaller than observed in the samples (data not shown). Ethanol was present in the cultures at concentrations 0.5–0.7 g/L, but it did not alter the pH because it does not undergo ionization.

In ILM at pH 7.0, growth curves of each strain were very different (Fig. 4A, middle panel). MYA-796 grew relatively slowly at the beginning, but ethanol and acetate detected as early as 24 h (Fig. 4F). At 48 h, glucose was no longer present and pH dropped to 5.9. This sample was collected at the beginning of logarithmic growth, which started at 45 h and flattened after 65 h. During this stage, ethanol and acetate were used and by the end of cultivation, they were both depleted, whereas pH rose again. Biocodex grew at a slower rate than MYA-796. At 60 h, the slope of the growth curve began to change, which could indicate entry into logarithmic phase, although this was not fully observed before the end of the experiment. In the sample taken at 48 h, glucose was no longer present, but acetate was detected for the first time and its concentration increased further at 72 h. The pH declined to 5.8 by the end of the culture. Ethanol was first detected at 24 h, reached 0.59 g/L at 48 h, and then started to decline slowly. NCYC3264 depleted all the glucose and reached a plateau before 30 h of cultivation, with Green Values close to 10. No further changes were observed in pH and acetate or ethanol content. Compared to other *S. boulardii* strains, this one produced more ethanol, up to 0.85 g/L.

In ILM at pH 7.7, the curves for Biocodex and MYA-796 increased until the end of cultivation (Fig. 4A, right panel. In both cases, the pH decreased slowly throughout the experiment. Biocodex did not use all the glucose before the end of the experiment, while acetate appeared for the first time at 48 h. Green Values of MYA-796 reached 14.2 ± 0.3. Glucose utilization was slower than in more acidic ILM, and traces were still detected at 48 h. Acetic acid was already present at 24 h, and its concentration increased until the end of the experiment. For NCYC3264 (Fig. 4G), the maximum Green Value was only 4.2 ± 0.1. The growth curve reached a plateau at 38 h, and no further changes in Green Values were recorded between 48 and 70 h under both pH values and culture broth compositions. Glucose was not fully utilized, while acetate (0.15 g/L) and ethanol (0.79 g/L) remained at similar levels.

### Quantification of robustness

The results of all experiments, in which YPD was used for biomass production, served to evaluate the robustness of *S. boulardii* strains. Microbial robustness is defined as the ability to maintain consistent performance despite external or internal perturbations. We used the Fano factor-based formula for calculating robustness (Eq. 1) [22]: values were in the negative range, and those closest to zero indicated the most robust strains.

The key issues when assessing robustness are proper strain selection and the perturbation space applied. Perturbation space is usually defined as a set of perturbations that occur during cultivation. However, the specifics of the present work required a modification of this definition. The cultivations were performed under only a few pH values, but each strain was subjected to a wide variety of conditions during initial biomass production. Thus, even though values used to calculate robustness were measured during cultivation (Step 3), the perturbations influencing performance appeared before that (Step1).

**Fig. 5:**
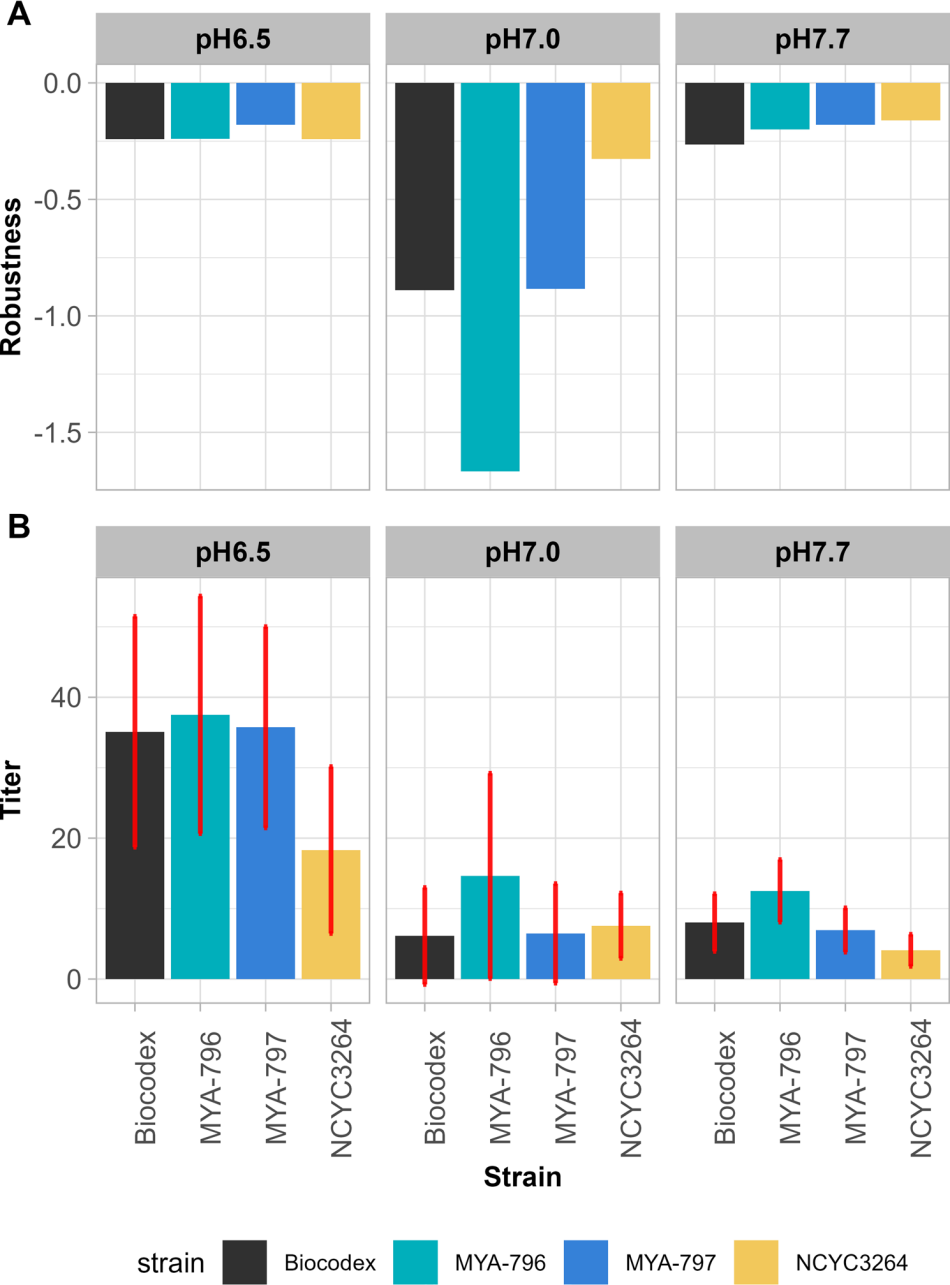
Evaluation of robustness in *S. boulardii* strains with respect to the titers obtained in ILM at pH 6.5, 7.0, and 7.7. **A** Robustness. **B** Titers. Bar plots denote the mean ± standard deviation (calculated for all sets of experiments for a strain in chosen pH)

When assessing robustness with regard to titer, i.e., the maximum Green Values achieved during cultivation at the selected pH, the Fano factor was calculated separately for cultures grown at each pH (Fig. 5A). For pH 6.5 and 7.7, the robustness results were similar and close to zero for all *S. boulardii* strains. Instead, at pH 7.0, robustness values were much more negative, and differences between the strains became apparent. The most robust strain under these conditions was NCYC3264, the least robust was MYA-796.

To understand the low robustness values at pH 7.0, we looked at the mean and standard deviation of titers (Fig. 5B) that were substituted into Eq. 2. In the case of cultivations at pH 6.5 and 7.7, robustness values were similar across strains, but the titers were different. At pH 6.5, each strain exhibited a high average titer, but also a high standard deviation. At pH 7.7, titers and standard deviations were contained. It should be noted that the means and standard deviations for each strain were calculated across the entire experimental set (with different sources of biomass) rather than for individual trials, thus the standard deviations are inherently higher. Robustness values were lower in ILM at pH 7.0, because standard deviations were very large compared to the low average titers. Strain MYA-796 attained consistently the best titers, but also the largest standard deviation, explaining why it was the least robust. In our set-up, low robustness indicated greater strain flexibility. Strain MYA-796 was more susceptible to modifications in biomass production; however, that may result in improved survival and growth under the erratic gut-like conditions.

## Discussion

In this work, we investigated the influence of production method on the functionality of probiotic yeasts during exposure to gastrointestinal -like conditions. Using this approach, we determined how yeast quality depended on biomass processing.

### Focus on the colon as a challenging environment for yeast

The model of the gastrointestinal tract recreated the physical and chemical conditions, but left aside interactions with other microorganisms or the host epithelium. This approach allowed us to focus on the physiology of *S. boulardii*. As in other works on yeast probiotics, we subjected the cells to stressors typical of the stomach and small intestine but expanded the study to develop and include also conditions mimicking the large intestine.

To select a suitable medium for cultivation, we started with ILM because it was previously used to study the transcriptome of *S. boulardii* [23]. This predefined medium contained a high concentration of bile salts and digestive enzymes and simulated the conditions in the small intestine. The tested *S. boulardii* strains could survive several hours of incubation but proved incapable of growth in such a high concentration of bile salts (data not shown).

To imitate the conditions of the large intestine, ILM without bile salts and digestive enzymes was prepared. The medium contained only 2.4 g/L glucose, which is eight times less than typical yeast media, and the main source of both carbon and nitrogen were amino acids derived from tryptone (5.7 g/L). Interestingly, glycerol production was observed in all ILM cultures (Fig. 4) and once generated, its concentration remained constant. Glycerol production by *S. cerevisiae* is associated primarily with response to osmotic stress [27], but may serve also to counteract a redox imbalance [28], toxic compounds elevated temperature[29] or multiple stressors [30]. The concentration of NaCl in ILM was 6.4 g/L, which was too low to activate the osmotic stress response [31]. There were no inhibitory compounds in the initial medium, but use of amino acids as carbon source might lead to the formation of harmful by-products [5]. Alternatively, glycerol production could function as a metabolic strategy to mitigate redox imbalance under hypoxic conditions by facilitating the essential reoxidation of cytosolic NADH to NAD⁺ [25, 26]. Hence, glycerol was likely an indicator of a general response to combinations of various stressors [29] and limited access to oxygen [28].

Monitoring glycerol and ethanol production might provide insights into oxygen availability during the cultivation in the Growth Profiler. Although anaerobic plate covers were employed, the reutilization of ethanol produced during the initial hours of cultivation (observed in ILM pH6.5 – Fig. 4B) indicates that residual oxygen remained sufficient to support respiratory metabolism. However, the synthesized glycerol was not re-consumed, indicating a persistent redox imbalance and limited oxygen availability. Collectively, these findings point toward the existence of microaerobic conditions. From the perspective of developing the model of digestive track, this result is satisfactory; as it reflects the physiological reality that the human intestine is not a strictly anaerobic environment, as oxygen continuously diffuses from the subepithelial capillaries through the epithelial cells into the peripheral layer of the gut [32].

### Optimization of biomass production can improve the quality and properties of yeast cells

To improve the quality of probiotics, we investigated the importance of the incubation step. An experiment omitting this step (Fig. 1C), indicated that *S. boulardii* strains were unable to grow in ILM at pH 7.0 when weakened by exposure to stomach-like and small intestine-like stress conditions. This provided a starting point for optimizing production and obtain cells of better quality.

Transferring biomass production from 30 to 37 °C proved crucial, as it offered preadaptation to the higher temperature used in the gastrointestinal tract model. However, the change of temperature alone did not guarantee better survival. Another important factor was the growth phase in which cells were harvested. Early stationary phase, reached after 18 h of biomass production, proved to be the most favorable (Fig. 2). Stress resilience of *S. cerevisiae* cells in early stationary phase has been documented previously[33, 34] and has been associated with accumulation of protective compounds such as trehalose, thicker cell wall or entering a quiescent state [30, 31]. Quiescence is a reversible, non-dividing state that yeast cells enter when nutrients become limiting. Quiescence cells are more resilient to stress, which might have been an advantage during incubations in low pH and with bile salts. On the other hand, the longer the cells stay in quiescence state, the more difficult it is to come back to re-enter the cell cycle and resume the divisions [35]. This might explain why biomass harvested after 42 h in 37 °C performed worse compared to the batch harvested after 18 h.

Optimization of production conditions enabled yeast to grow in ILM at pH 7.0 (Fig. 4B). In the case of MYA strains, the growth phase comprises two stages. The first one was non-logarithmic and was accompanied by a decrease in pH. The second one was logarithmic, but appeared only after more than 40 h, which implies the possible change to a pH range more suitable for *Saccharomyces*.

HPLC measurements indicated that the lower pH in all ILM correlated with production of acetate (Fig. 4). However, once the acetate was reutilized, the pH increased again. Acetic acid has antibacterial and anti-inflammatory properties, and its presence in the gut is considered desirable. Previous studies on *S. boulardii* attempted to increase acetic acid production by screening the best strains [36], laboratory adaptation[37] or genetic modification [3]. In these studies, rich media with much higher glucose concentrations [3, 20, 36], 40 g/L[36] and even 200 g/L [37] were employed. Moreover aerobic conditions were reported as crucial to produce high levels of acetic acid [3]. In our system, we detected acetate produced by non-modified strains under oxygen-limited conditions and very low glucose concentrations (2.4 g/L), which could indicate that strains more specialized in acetate production could produce even larger amounts.

### Potential flexibility of probiotic strains

We conducted a robustness assessment, in which we examined how changes in biomass production affected the performance of *S. boulardii* strains under conditions resembling the gastrointestinal system. Robustness is defined as the ability to maintain consistent performance despite external or internal perturbations.

The most robust *S. boulardii* strain turned out to be the one with the worst performance (NCYC3264). Instead, the least robust strain (MYA-796) was the most susceptible to optimization of production conditions. The observed flexibility of MYA-796 is an interesting and potentially useful trait.

The optimizations we carried out allowed yeast to grow within the full range of pH values encountered under colon-like conditions, which might theoretically translate into an improved ability to colonize the large intestine. Colonization is considered one of the positive features of probiotics. However, would such a product always be the best one? Given the rare incidence of probiotic-related fungemia, increased sensitivity to a certain pH could be a desirable feature when designing probiotics for immuno-compromised individuals. Inhibition of yeast growth by a gradual increase in pH along the length of the large intestine could potentially protect against the occurrence of yeast infections. Thus, by changing production settings, it might be possible to obtain products tailored to the needs of different groups of consumers.

## Conclusion

In the present study, we developed a simple in vitro model of the gastrointestinal system, which allowed us to discriminate between strains of *Saccharomyces* of varying quality. The model could be a useful tool for further studies on the physiology or production of *S. boulardii*.

Existing efforts have tried to improve *S. boulardii* via genetic modification. However, in many countries such strains could not be used due to restrictions on genetically modified organisms. Here, we show that the properties of probiotic yeasts could be easily tailored by optimizing biomass production settings. We have omitted issues related to freeze-drying or long-term storage. These additional challenges make it even more important to ensure the highest quality of probiotics through optimized production processes.

## Supplementary Information

Supplementary Material 1.

## Data Availability

The datasets used and analyzed during the current study are available from the corresponding author on reasonable request.
