# Supplementary material for: Effect of the biomass production conditions on survival of the probiotic yeast Saccharomyces cerevisiae var. boulardii in a laboratory model of the gastrointestinal tract
Source: Microb Cell Fact. 2026 Apr 12;25:103. doi: 10.1186/s12934-026-02999-8 (PMC13088855; doi:10.1186/s12934-026-02999-8)
Supplement: Supplementary file 1 — Supplementary Material 1. [file 12934_2026_2999_MOESM1_ESM.docx]

**Supplementary File**

Experiments in the study are based on cultures grown in the high-throughput Growth Profiler plate reader. The system captures images at regular intervals and calculates Green Values (although options for Red and Blue Values are also available). We also generated calibration curves to estimate Cell Dry Weight (CDW) (Fig. S1). The *S. boulardii* MYA-796 strain was grown under four distinct conditions:

1. 30 °C in YPD medium, cells harvested in the exponential phase;
2. 37 °C in YPD medium, cells harvested in the exponential phase;
3. 30 °C in ILM (pH 7.7) medium, cells harvested in the stationary phase;
4. 37 °C in ILM (pH 7.7) medium, cells harvested in the stationary phase.

Samples were centrifuged and resuspended in ILM at pH 7.7. A portion of the biomass was used for dry mass measurements (filtration, drying, and weighing), while the remainder was used to prepare dilutions for Green Value (GV) measurements in the Growth Profiler.

The Green Values (GV) used to generate the calibration curve were obtained by subtracting the background signal of the sterile ILM pH 7.7 medium.

Although slight variations exist between the resulting curves, they remain remarkably similar considering the diverse conditions used for biomass preparation. Nevertheless, we decided to use Green Values in this study rather than converting them to CDW. Our rationale is as follows:

- Calibration inherently introduces error, as it is impossible to predict every environmental factor that might influence cell morphology.
- Furthermore, comparing different strains would require separate calibration curves for each, introducing additional measurement variability.
- To eliminate these potential errors, we prefer to compare raw data. Basing experiments on arbitrary units is a widely accepted practice, similar to standard OD_600_ measurements.

The Growth Profiler also offers a distinct advantage over other plate readers: the ability to visually verify whether growth curves result solely from cellular proliferation. An example is provided in Figure S2, which shows two images captured by the system after 48 hours of MYA-796 cultivation in ILM media at various pH levels. In the plate on the right, bile salts were present at a concentration of 1 g/L. We observed that these salts precipitated in media with a pH below 6. The resulting aggregates are clearly visible in the wells highlighted by the red rectangle.

**Figure S1**. Calibration curves for *S. boulardii* MYA-796 correlating Cell Dry Weight (CDW) with Green Values (GV). Curves were generated for four distinct growth conditions (30°C and 37°C in YPD or ILM pH 7.7) to evaluate the impact of cellular morphology on biomass estimation. GV data were baseline-corrected by subtracting the background signal of the sterile medium.


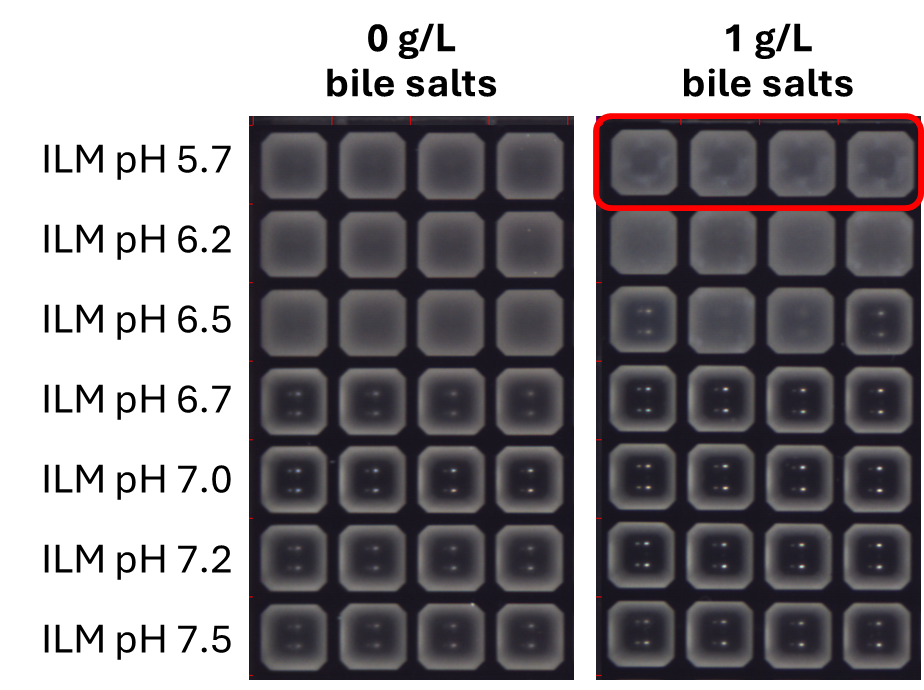


**Figure S2.** Photos of the made by Growth profiler of *S. boulardii* MYA-796 growth in Growth Profiler plates after 48 h of cultivation. Plates contain ILM media at various pH levels. The plate on the right was supplemented with bile salts, which precipitate under low pH conditions. This precipitation is highlighted by the red rectangle.
